# Measuring Health Outcomes in HIV: Time to Bring in the Patient Experience

**DOI:** 10.5334/aogh.2958

**Published:** 2021-01-05

**Authors:** Niki O’Brien, Y-Ling Chi, Karolin R. Krause

**Affiliations:** 1 Global Health and Development Group, Imperial College London, London, United Kingdom; 2 Institute of Global Health Innovation, Imperial College London, London, UK; 3 Center for Global Development Europe, London, United Kingdom; 4 Anna Freud National Centre for Children and Families, London, United Kingdom

## Abstract

**Introduction:**

Over the past decade, the global response to HIV has led to a reduction in the number of new infections, and a decrease in associated mortality. Yet, the number of people living with HIV (PLHIV) is high, with an estimated 38 million infected worldwide. As HIV shifts from being an acute terminal illness to a chronic condition, evaluating programmatic responses to HIV with sole reliance on biological markers (such as viral load or CD4 cell count) as proxies for patient health may no longer be suitable. HIV affects the lives of those infected in myriad ways which should be reflected in programme evaluations by measuring health-related quality of life, in addition to biomarkers.

**Discussion:**

In this commentary we argue that there is a pressing need to review how a “good” health outcome is defined and measured in light of care systems moving towards value-based frameworks that measure value in terms of the actual health outcomes achieved (rather than processes of care), global response shifting to providing long-term care for PLHIV in the community, and integrating HIV as part of universal health coverage plans. Efforts should be directed towards validating generic and disease specific patient-reported measures of PLHIV, to identify the most suitable tools. Such efforts will ensure that patient experience is appropriately captured, especially to be used in programme or economic evaluations.

**Conclusions:**

It is only by recognising and measuring the full range of health, mental and social outcomes related to the disease that the health status of PLHIV can be fully understood.

## INTRODUCTION

Over the past decade, the global response to HIV has yielded a reduction in the number of new infections, and a decrease in associated mortality. Yet, the number of people living with HIV (PLHIV) is high, with an estimated 38 million infected worldwide [1]. Programme evaluation plays a crucial role in monitoring whether designated interventions succeed at improving health outcomes for PLHIV. There is a pressing need in economic evaluation to review how a “good outcome” is defined and measured as care systems move towards value-based frameworks that determine value in terms of health outcomes achieved rather than programme inputs delivered per dollar spent [2]; and as the global response shifts from prevention and disease suppression towards providing long-term care for PLHIV.

Viral load (VL) is a key indicator of HIV treatment success [3]. VL measures the number of copies of HIV RNA per millilitre of blood, with a low VL indicating viral suppression. VL is frequently reported as a primary outcome measure, often in combination with CD4 cell count, which measures the number of CD4 immune cells – an essential part of the human immune system – per cubic millimetre of blood. CD4 cell count thus assesses immune system response and risk of opportunistic infections. During the decades when HIV represented a terminal illness, and hospital-based care for HIV patients dominated the global response, these biomarkers provided an effective way of monitoring treatment impact, and of informing individual treatment plans [4,5]. Today, VL and CD4 cell count are still preferred indicators of success for payers, and often the sole metric of patient health when assessing intervention or program value (including cost-effectiveness).

Over the past two decades, however, the global HIV response has changed. Coverage of antiretroviral therapy (ART) has expanded steadily, with the Joint United Nations Programme on HIV and AIDS (UNAIDS) aiming for 81% coverage by 2020 [6]. As a result, people with HIV are living longer: the life expectancy of those who maintain viral suppression on ART is now similar to that of individuals without HIV, and the global population aged 50 or older living with HIV has increased [7]. As HIV shifts towards becoming a chronic rather than a terminal disease, models of care are moving away from hospitals, towards integrated, primary care or community based approaches [4,8,9].

### ARE WE MEASURING WHAT MATTERS?

While life expectancy of PLHIV is increasing, treatment does not fully restore immune health. HIV is associated with an increased risk for complications such as cardiovascular disease and cancer, and PLHIV have reported lower health-related quality of life (HRQoL) than the general population, even when the virus is successfully supressed and the immune system stabilised [4,5,10]. Virally suppressed patients are more likely to report symptoms such as fatigue, lack of energy, insomnia, depression, and sexual dysfunction [11]. Recent research from the United Kingdom (UK) suggests that PLHIV and healthcare and service providers are concerned with a broad range of physical, psychological, cognitive, and socio-economic outcomes, such as, change in pain and gastrointestinal symptoms, anxiety and depression, financial or legal issues [12]. Shifts towards HIV as a chronic disease, paired with the broad range of outcomes considered important by key stakeholder groups, raise questions about whether biomarkers such as VL and CD4 cell count are still sufficient stand-alone indicators to determine value in HIV treatment. While they are excellent metrics of disease state, they do not provide a broader view of health, defined by the World Health Organization (WHO) as “a state of complete physical, mental and social well-being, and not merely the absence of disease [13].”

### MEASURING HEALTH-RELATED QUALITY OF LIFE IN PLHIV

HRQoL describes all “those aspects of self-perceived well-being that are related to or affected by the presence of disease or treatment,” across physical, mental and social domains of health [14]. Measures of HRQoL could provide a more comprehensive picture of how treatment affects a patient’s life when used alongside clinical and biological markers in economic evaluations of HIV interventions. HRQoL is recommended for inclusion in clinical trials for a range of other chronic conditions, including type-2 diabetes [15], osteoarthritis and Crohn’s disease [16,17], by so-called core outcome sets that provide standards for outcome measurement and were developed jointly with patients. Similarly, a consultation of patients and carers in relation to adult epilepsy found that 86% and 92%, respectively, considered quality to life to be an important outcome to measure in epilepsy trials [18]. In programme and economic evaluations, HRQoL measures have been rolled out as part of randomized or quasi-experimental research designs, e.g. oftentimes by comparing health outcomes achieved in the intervention group with outcomes achieved in a matching control group. Such research designs can help control for confounders that may influence HRQoL measurement.

HRQoL can be measured using generic or condition-specific measures. Generic measures enable comparisons and benchmarking across different conditions, such as for cost-effectiveness analyses, by enabling the calculation of Quality Adjusted Life Years (QALYs). However, they may fail to capture subtle, condition-specific changes in wellbeing. In turn, disease-specific measures are designed to assess a condition’s specific impact on HRQoL. Being more tailored to patient concerns, these measures may be perceived as more meaningful and acceptable in clinical and programmatic settings, and provide superior sensitivity to change [19]. They also facilitate the attribution of changes in HRQoL to changes in the underlying disease, rather than the influence of external factors.

The European Quality of Life-5 Dimensions (EQ-5D) is the most commonly used generic HRQoL measure in HIV research and has the most extensive psychometric evidence base [5,20]. EQ-5D comprises five questions on mobility, self-care, pain, usual activities, and psychological status. Respondents indicate their level of difficulty in each health dimension, and an overall index of health state can be obtained [21]. By applying population-specific weights (i.e., so-called value sets) to each health dimension, this index can summarize the quality of a person’s health according to cultural preferences in a given country and be used to calculate QALYs for the purpose of economic evaluations. Wu and colleagues found that EQ-5D was administered in only five HIV trials [22], during the period of 2001 to 2010. While the EQ-5D lends itself well to use in cost-effectiveness analysis, it has shown ceiling effects in PLHIV, with around 40% of patients obtaining the highest possible score, denoting excellent health, according to one review [5].

In contrast, the Medical Outcomes study HIV Health Survey (MOS-HIV) is the most widely used and validated HIV-specific HRQoL measure [5,22,23]. It consists of 35 items across 11 domains of health and wellbeing. While it is more comprehensive and more specific than the EQ-5D, the MOS-HIV also has limitations. While the English version of the questionnaire takes approximately 5–10 minutes to complete, it reportedly takes considerably longer to complete some of its translations [5]. In addition, there are a number of modified versions of the scale, that were adapted for use in different cultural contexts, leading to a lack of comparability [24]. Furthermore, scoring and interpretation are considered complex and may be difficult to integrate into programme evaluations [5]. Finally, MOS-HIV was adapted from existing generic measures, and may not fully capture the breadth and depth of disability experienced specifically by PLHIV, which may affect its sensitivity to change in this population [22]. Two more recently developed HIV-specific measures (i.e., the WHOQOL-HIV BREF and PROWOL-HIV) may offer viable alternatives to the MOS-HIV, but evidence on their psychometric properties is still limited [5]. As generic and disease-specific measures of HRQoL have different inherent strengths and disadvantages, they may be used alongside one another to form a comprehensive assessment [22].

### LOOKING AHEAD

Further research is needed to identify the most suitable approaches to measuring HRQoL in PLHIV for the purpose of programme or economic evaluation (including cost-effectiveness analysis). The use of such measures is particularly mandated for evaluating complex interventions such as Human Rights Interventions or service delivery redesign (e.g. differentiated care models), where consideration of non-clinical outcomes appears increasingly warranted. As countries move towards achieving universal health coverage, a metric for cross-disease comparisons is needed to allow Ministries of Health to compare the relative value of treatments within a disease area, and across diseases, in order to ensure they offer patients the best treatment options. This is another reason to shift away from the sole reliance on VL as a treatment outcome in HIV, and towards inclusion of a generic measure of HRQoL, such as EQ-5D. Finally, a greater focus on HRQoL could also inform the provision of person-centred care in routine clinical settings, although this is outside of the remit of this article.

In combination with suitable HRQoL measures, as well as biomarkers, a more complete picture of health and intervention value can be obtained. Within a value-based framework, it is critical that the patient voice is integrated into the process of identifying suitable measurement approaches alongside voices of other key stakeholder groups [25]. There are early stage initiatives within the HIV community: The British HIV Association has begun to explore the priorities of adults living with HIV, health care professionals, and commissioners to ensure outcome measurement tracks what matters most [12]. As noted previously, patients can be brought into the development of standards for meaningful outcome measurement, for example, through the development of consensus-based core outcome sets that provide a minimum standard of outcomes to be measured and reported by all those evaluating treatments for a specific condition [26]. The COMET Initiative, which maintains a register of core outcome sets developed worldwide, currently contains only one HIV-related entry in contrast to 50 core outcome sets for cancer, and close to 40 related to rheumatology [27]. There is scope for HIV researchers, practitioners, and patients to come together and develop a consensus-based approach to measuring treatment response for HIV that is comprehensive and meaningful to all key stakeholder groups.

To advance the agenda of more comprehensive and meaningful outcome measurement in HIV, more extensive validation efforts are needed to identify the instruments that offer a high degree of validity, reliability, and sensitivity to change, and are most suitable for cross-cultural use in practice settings. The EQ-5D-3L is available in over 180 languages [21], but other tools may need to be translated and validated for use in new contexts. Validation studies will need to assess the measurement invariance of these tools, that is, the extent to which the same construct of HRQoL is measured across different populations and subgroups. In addition, country-specific value sets for EQ-5D, which are essential to derive QALYs, are only available in a handful of Sub-Saharan Africa countries to date (e.g. Ethiopia and South Africa), which currently hinders wide-spread use of EQ-5D for economic evaluations in this region.

To accelerate development and validation efforts, researchers assessing cost-effectiveness of HIV interventions could consider adding HRQoL measures to patient surveys that are already part of ongoing evaluations or monitoring systems. A recent economic evaluation conducted in Kenya by one of the authors (YC) used EQ-5D and found this measure to have good acceptability amongst patients. In line with reported experience in other countries, the questionnaire was simple enough that it did not require lengthy instructions, and only a few minutes were spent on administration [28]. Adding this brief scale did not significantly increase the cost of data collection or analysis. The HIV community, including development partners such as the Global Fund or the President’s Emergency Plan for AIDS Relief (PEPFAR) could pilot HRQoL measurement as part of their evaluation strategies alongside traditional metrics such as VL. The UK National Health Service already generally uses generic and disorder-specific measures of HRQoL alongside one another to assess and benchmark patient health [29].

## CONCLUSIONS

By seizing opportunities to pilot the use of HRQoL measures in PLHIV, and by validating specific measures, lessons can be learned and shared globally, with the potential to move outcome measurement for PLHIV into the next decade. These lessons will be invaluable to those interested in developing a core outcome set for HIV, as this will require appraising the measures available for their psychometric property and feasibility.

A full understanding of what it means to be healthy in the context of HIV treatment requires a more holistic approach to the measurement of health. It is only by recognising and measuring the full range of associated health, mental and social outcomes related to the disease that the health status of PLHIV can be fully understood.
